# LOX-1 Regulates *P. gingivalis*-Induced Monocyte Migration and Adhesion to Human Umbilical Vein Endothelial Cells

**DOI:** 10.3389/fcell.2020.00596

**Published:** 2020-07-14

**Authors:** Qian Li, Jianru Liu, Wenyi Liu, Yi Chu, Jinsheng Zhong, Ying Xie, Xinzhe Lou, Xiangying Ouyang

**Affiliations:** ^1^Department of Periodontology, Peking University School and Hospital of Stomatology, Beijing, China; ^2^First Clinical Division, Peking University School and Hospital of Stomatology, Beijing, China

**Keywords:** *Porphyromonas gingivalis*, LOX-1, THP-1 cells, HUVECs, migration, adhesion

## Abstract

*Porphyromonas gingivalis* (*P. gingivalis*) is one of the main periodontal bacteria. This pathogen was reported to enhance monocyte migration and adhesion to endothelial cells in atherosclerosis. The scavenger receptor lectin-like oxidized low-density lipoprotein receptor-1 (LOX-1) plays a pivotal role in atherogenesis. The aim of this study was to investigate whether LOX-1 modulates *P. gingivalis*-mediated monocyte migration and adhesion to endothelial cells and how it works. The results showed that the migration and adhesion of monocytic THP-1 cells to human umbilical vein endothelial cells (HUVECs) were significantly enhanced when HUVECs or THP-1 cells were challenged with *P. gingivalis*. Meanwhile, the expression level of LOX-1 in both HUVECs and THP-1 cells were also significantly increased by *P. gingivalis* stimulation. It is well known that ligand/receptor pairs monocyte chemoattractant protein-1 (MCP-1)/CC chemokine receptor 2 (CCR2), selectins/Integrins, and cell adhesion molecules (CAMs)/Integrins mediate monocyte migration and adhesion to endothelial cells. In this study, LOX-1 was demonstrated to be crucially involved in *P. gingivalis*-induced THP-1 cell migration and adhesion to HUVECs, by regulating expression of ligands MCP-1, intercellular adhesion molecule-1 (ICAM-1) and E-selectin in HUVECs and that of their receptors CCR2 and Integrin αMβ2 in THP-1 cells. The nuclear factor-kappa B (NF-κB) signaling pathway was proved to be involved in this process. In conclusion, LOX-1 plays a crucial role in *P*. *gingivalis-*induced monocyte migration and adhesion to endothelial cells. This result implies LOX-1 may act as a bridge in linking periodontitis to atherosclerosis.

## Introduction

Atherosclerosis is the major etiology of cardiovascular disease, the leading cause of mortality worldwide (Mayer and Binder, 2019). The activation of endothelial cells, adhesion of monocytes, proliferation of smooth muscle cells, and formation of atherosclerotic plaques are the main pathological processes of atherosclerosis (Kaski, 2011; Theodorou and Boon, 2018). In atherogenesis, monocyte migration and adhesion to endothelial cells are crucial events. A tightly regulated multistep process involving ligand-receptor interactions is contributed to these events. Endothelial cells express various inflammatory molecules when they are activated by kinds of stimuli such as low-density lipoprotein, hyperlipidemia, low shear stress, and TNF-α (Mestas and Ley, 2008). These inflammatory molecules then interact with their receptors expressed in monocytes to mediate the recruitment, retention and firm adhesion of circulating monocytes. Among them, monocyte chemoattractant protein-1 (MCP-1) is a pivotal molecule which mobilizes monocytes by interacting with receptor C-C chemokine receptor type 2 (CCR2) (Bianconi et al., 2018). In addition, selectins and cell adhesion molecules (CAMs) interact with Integrins such as Integrin αMβ2, Integrin α4β1, and Integrin αLβ2, to regulate the subsequent rolling and firm adhesion of monocytes (Jaipersad et al., 2014). Therefore, the expression of MCP-1, selectins, CAMs and their receptors is extremely crucial for monocyte migration and adhesion to endothelial cells. After migrating and adhering to endothelial cells, monocytes would emigrate into the intima and become lipid-laden macrophages, which contribute to the formation of atherosclerotic plaques (Jaipersad et al., 2014).

Lectin-like oxidized LDL receptor-1 (LOX-1), a class E scavenger receptor belonging to the family of pattern recognition receptors (PRRs), was reported to play a critical role in atherosclerosis. It was originally identified in endothelial cells, and subsequently found to be expressed in other cells such as macrophages, smooth muscle cells (SMC), and platelets (Nvk et al., 2017). LOX-1 expression is at a low level under physiological conditions but can be upregulated by pathogens, oxidized low-density lipoprotein (ox-LDL), and proinflammatory stimuli such as the cytokines tumor necrosis factor α (TNF-α) and transfer growth factor β (TGF-β) (Navarra et al., 2010). Once activated, LOX-1 can initiate a cascade of intracellular signaling pathways, mainly involving MAPK, PI3K/Akt, JNK, PKC, NF-κB, PPAR-γ pathways, finally resulting in endothelial cell dysfunction, monocyte migration and adhesion, SMC growth and migration, and platelet aggregation, which are all key steps in atherogenesis (Zhu, 2005; Lu et al., 2011).

Periodontitis is a bacteria-initiated disease of the periodontium (Cardoso et al., 2018). This oral disease is the sixth most prevalent disease worldwide, affecting approximately 50% of the global population (West, 2019). It has been confirmed to be associated with an increased risk of atherosclerosis by multiple epidemiological and interventional studies. *Porphyromonas gingivalis* (*P. gingivalis*), which serves as a keystone pathogen in periodontitis, is implicated in various processes of atherosclerosis by direct invasion or systemic cytokine release (Amar and Engelke, 2015). Although *P. gingivalis* has been demonstrated to enhance monocyte migration and adhesion to endothelial cells (Hashizume et al., 2011; Zhou et al., 2011), the exact mechanisms are less well understood.

LOX-1 is reported to recognize bacteria such as *S. aureus*, *Escherichia coli* (Shimaoka et al., 2001) and *Chlamydia pneumonia* (Campbell et al., 2013). However, no studies have focused on the relationship between LOX-1 and periodontal pathogen *P. gingivalis* yet. Whether LOX-1 modulates the *P. gingivalis*-mediated monocyte migration and adhesion to endothelial cells also remains unexplored. Our study was designed to investigate whether and how LOX-1 modulates *P. gingivalis*-mediated monocyte migration and adhesion to endothelial cells.

## Materials and Methods

### Cell Culture

Human umbilical vein endothelial cells (HUVECs) and human monocytic THP-1 cells were purchased from ScienCell (California, United States). HUVECs were cultured in Endothelial Cell Medium (ECM; ScienCell), supplemented with 10% fetal calf serum (FBS; ScienCell) and 200 ng/ml endothelial cell growth supplements (ECGs; ScienCell) at 37°C with 5% CO_2_ in a humidified atmosphere. HUVECs from passages 3–6 were used for all experiments. THP-1 cells were maintained in RPMI 1640 medium (HyClone, Logan, UT, United States) containing 10% FBS (ScienCell) in a humidified atmosphere (37°C, 5% CO_2_).

### Small Interfering RNA Transfection and Lentiviral Transduction

LOX-1-specific small interfering RNAs (siRNAs) including siLOX-1-1, siLOX-1-2, and siLOX-1-3 and non-targeting control siRNA (scrambled) were synthesized by Sangon Biotech (Shanghai, China). The sequences of siRNAs are listed in Table 1. The LOX-1-specific siRNAs and control siRNA (20 nM) were transfected into HUVECs and THP-1 cells using Lipofectamine3000^®^ reagent (Invitrogen, Carlsbad, United States), according to the manufacturer’s instructions. After 48 h, Western blotting was applied to validate the effectiveness of RNA interference (RNAi).

**TABLE 1:** RNAi sequences used in this study.

RNAi	Sense strand (5′–3′)	Antisense strand (5′–3′)
siRNA		
siLOX-1-1	CCAUUAUGGUGCUGGGCAUTT	AUGCCCAGCACCAUAAUGGTT
siLOX-1-2	GGAAAUGAUAGAAACCCUUTT	AAGGGUUUCUAUCAUUUCCTT
siLOX-1-3	GCAAGACUGGAUCUGGCAUTT	AUGCCAGAUCCAGUCUUGCTT
scrambled	UUCUCCGAACGUGUCACG UTT	ACGUGACACGUUCGGAGAATT
shRNA		
LV-shLOX-1	CCCTTCAGGTACCTGTGCATATATA	
LV-Con2	TTCTCCGAACGTGTCACGT	

Lentiviruses encoding the LOX-1 gene (LV-LOX-1) or containing a LOX-1-specific short hairpin RNA (LV-shLOX-1) as well as their respective controls (LV-Con1 and LV-Con2) were constructed by GeneChem (Shanghai, China). Their sequences are shown in Table 1. HUVECs and THP-1 cells were transduced with the lentiviral particles at a multiplicity of infection (MOI) of 10:1 in the presence of 10 ng/ml polybrene (Sigma-Aldrich, St Louis, MO, United States). The virus-containing medium was removed after 24 h and replaced with fresh medium. The transduction efficiency was assessed by Western blotting at 72–96 h after lentiviral transduction.

### Bacterial Culture

*Porphyromonas gingivalis* strain W83 was a gift from Prof. Chenxiong Lai at Kaohsiung University. The *P. gingivalis* was grown for 4–6 days on brain heart infusion (BHI) blood agar plates (BD Biosciences, California, United States) which contained 5% defibrinated sheep blood, 5 mg/ml yeast extract, 5 μg/ml hemin, and 1 μg/ml vitamin K1 (Sigma-Aldrich) in an anaerobic system (10% H_2_, 85% N_2_, and 5% CO_2_) at 37°C. Bacterial colonies were then inoculated into BHI broth medium supplemented with 5 μg/ml hemin, and 1 μg/ml vitamin K1, and cultured for 24 h. The bacteria were then harvested by centrifugation (6000 rpm, 4°C, 10 min), washed with phosphate buffered salt solution (PBS, PH = 7.2), and resuspended in antibiotic-free cell medium. Bacterial resuspension was adjusted to an optical density (OD) of 0.5 at 600 nm, corresponding to a concentration of 10^8^ CFU/ml.

### Bacterial Challenge

Bacterial challenge assay was conducted as previously described (Wan et al., 2015). Briefly, the prepared bacterial resuspension was added to HUVECs monolayers or to THP-1 cells at a MOI of 100:1 for 2 h, after which the medium was replaced with fresh medium containing 0.5 mg/ml gentamicin and 0.1 mg/ml metronidazole (Zhongshan Golden Bridge, Beijing, China). Subsequently, the HUVECs and THP-1 cells were cultured for indicated times. The duration of *P. gingivalis* stimulation was the sum of these two periods. This treatment has been shown not to affect the viability of cells (Wan et al., 2015).

### Inhibition of NF-κB Signaling Pathway

HUVECs and THP-1 cells were preincubated, respectively, with ammonium pyrrolidinedithiocarbamate (PDTC; Sigma-Aldrich), an inhibitor of NF-κB activation, at a concentration of 100 μM for 1 h before they were further challenged with *P. gingivalis* for 24 h. Likewise, LOX-1-overexpressing HUVECs and LOX-1-overexpressing THP-1 cells were treated with PDTC (100 μM) for 24 h before cells were harvested.

### Migration Assay

The impact of *P. gingivalis* on migration of THP-1 cells toward HUVECs was determined using 24-well transwell systems (8-μm pore size; Corning, New York, United States).

On one hand, untreated HUVECs, HUVECs with or without LOX-1 knockdown (si-LOX-1 and scrambled, respectively), as well as LOX-1-overexpressing (LV-LOX-1) and control (LV-Con1) HUVECs (2 × 10^5^ cells/well) were seeded, respectively, in the lower compartments containing ECM with 10% FBS to form confluent monolayers. Among them, the untreated HUVECs, and HUVECs with or without LOX-1-knockdown were challenged with *P. gingivalis* for 24 h or left untreated. At the same time, untreated THP-1 cells were labeled with calcein AM (Thermo Fisher, Waltham, MA, United States) or Hoechst 33342 (Sigma-Aldrich) for 30 min at 37°C, according to the manufacturer’s instructions, before being resuspended in RPMI 1640 medium (HyClone) with 10% FBS. Then, the labeled THP-1 cells were plated into the upper inserts (1 × 10^5^ cells/well) and incubate with the primed HUVECs monolayers for 6 h at 37°C.

On the other hand, untreated HUVECs were seeded at a density of 2 × 10^5^ cells per well in the lower compartments containing ECM with 10% FBS to form confluent monolayers. Untreated THP-1 cells, and THP-1 cells with or without LOX-1 deficiency (si-LOX-1 and scrambled, respectively) were challenged with *P. gingivalis* for 24 h or left untreated. These THP-1 cells as well as LOX-1-overexpressing (LV-LOX-1) and control (LV-Con1) THP-1 cells were labeled with calcein AM (Thermo Fisher) or Hoechst 33342 (Sigma-Aldrich), and resuspended in serum free RPMI 1640 medium, respectively. They were then added into the upper chambers at a concentration of 1 × 10^5^ cells/well and incubated with the monolayers of HUVECs in the lower chambers for 6 h at 37°C.

The fluorescence microscopy (Nikon, Tokyo, Japan) was applied to visualize and capture THP-1 cells entering into the lower chambers. Migrated THP-1 cells were counted from five random fields of view and quantified by Image J software (NIH, Bethesda, United States).

### Adhesion Assay

Adhesion assay was performed in 24-well plates to measure the impact of *P. gingivalis* on adhesion of THP-1 cells to HUVECs.

On one hand, untreated HUVECs, LOX-1-knockdown (si-LOX-1) and control (scrambled) HUVECs, as well as LOX-1-overexpressing (LV-LOX-1) and control (LV-Con2) HUVECs (2 × 10^5^ cells/well) were seeded, respectively, in plates containing ECM with 10% FBS to form confluent monolayers. The untreated HUVECs, LOX-1-knockdown and control HUVECs were challenged with *P. gingivalis* for 24 h or left untreated. At the same time, according to the manufacturer’s instructions, untreated THP-1 cells were labeled for 30 min with Hoechst 33342 (Sigma-Aldrich) at 37°C. They were then resuspended in ECM with 10% FBS and seeded at a density of 1 × 10^5^ cells/well onto the primed HUVECs monolayers, respectively, followed by incubation for 2 h at 37°C.

On the other hand, the 24-well plates were seeded with untreated HUVECs at 2 × 10^5^ cells per well to form confluent monolayers. Untreated THP-1 cells, LOX-1 deficient and control THP-1 cells were challenged with *P. gingivalis* for 24 h or left untreated. These THP-1 cells as well as LOX-1-overexpressing and control THP-1 cells were labeled with Hoechst 33342 (Sigma-Aldrich) and resuspended in ECM with 10% FBS, respectively. After that the prepared THP-1 cells were plated onto the HUVECs monolayers and incubated for 2 h at 37°C.

Non-adherent THP-1 cells were gently washed with PBS, and the adherent THP-1 cells were imaged with a fluorescence microscope (Nikon, Tokyo, Japan). The number of adherent THP-1 cells were counted in at least five randomly selected areas and quantified using Image J software (NIH).

### RNA Extraction and Real-Time PCR Analysis

Total RNA was extracted from cultured cells using TRIzol reagent (Sigma-Aldrich). 1 μg aliquots of RNA from each sample were reverse transcribed into cDNA using the PrimeScript RT reagent Kit (TaKaRa, Kusatsu, Japan). SYBR^®^ Premix Ex Taq (Takara) was used to perform Real-time PCR as described previously (Liu et al., 2018). The expression of target genes was quantified by the comparative 2^–Δ^ ^Δ^ ^Ct^ method after normalization to glyceraldehyde-3-phosphate dehydrogenase (GAPDH) expression. The primers specific to LOX-1, MCP-1, ICAM-1, E-selectin, Integrin αM, Integrin β2, CCR2, and GADPH are listed in Table 2.

**TABLE 2:** Sequences of primers used for real-time PCR.

Genes	Primer sequences (5′–3′)
LOX-1	Forward: GCAGAAGAAGCTTCACAGGAGTCAG
	Reverse: TGGAGATTCAGATTCTGGTGGTGAAGT
MCP-1	Forward: GAAAGTCTCTGCCGCCCTT
	Reverse: TTGATTGCATCTGGCTGAGCG
ICAM-1	Forward: TGAGGAGAGATCACCATGGAGC
	Reverse: GCCAGGGAACAGACCACGGT
E-selectin	Forward: CCCTATGCTACACAGCTGCC
	Reverse: GCTTCCATGCTCAGGGGATT
Integrin αM	Forward: CACGCAGACAGACACAGGTC
	Reverse: TCCCGAAAGCAGACAATGGC
Integrin β2	Forward: CACCATGTCTGCCCCATCAC
	Reverse: GCTGTCATTTTGAGGGCGGA
CCR2	Forward: TCCGAAAGCACATCACCAAGCG
	Reverse: CCCCAGTGGAAGGCGTGTTTG
GADPH	Forward: GTGACCAGGCGCCCAATAC
	Reverse: CGTCGCCAGCCGAGCCACA

### Western Blotting

Total cellular proteins were extracted from cultured cells using the protein extraction kit (Solarbio, Beijing, China). An equal amount of protein (40 μg) from each sample was subjected to SDS-PAGE and then transferred onto polyvinylidenedifluoride (PVDF) membranes (Thermo Fisher). After blocking with 5% skimmed milk at room temperature for 1 h, the membranes were incubated with primary antibodies at 4°C overnight followed by incubation with horseradish peroxidase (HRP)-conjugated anti-rabbit or anti-mouse secondary antibodies (1:10000, ZSGB-Bio, Beijing, China) for 1 h at room temperature. Finally, the protein bands were imaged with Chemidox XRS (Bio-Rad, Hercules, United States) and quantified with Quantity One software (Bio-Rad). The following primary antibodies were used: LOX-1, ICAM-1, E-selectin, Integrin αM, Integrin β2 (1:1000, Bioss, Beijing, China), CCR2 (1:200, Santa Cruze, Delaware Ave, United States), MCP-1, p65, p–p65 (1:1000, Abcam, Cambridge, United Kingdom) and GADPH (1:1000, Protein, Wuhan, China).

### Statistical Analysis

The statistical analysis was performed using GraphPad Prism 7.0 software (GraphPad Software Inc, La Jolla, United States). Unpaired two-tailed Student’s *t*-test for two groups was used. Analysis of variance (ANOVA) followed by Dunnett’s multiple comparison test or Turkey’s multiple comparison test for multiple groups was carried out. Results are presented as the mean ± standard deviation (SD) from at least three independent experiments. ^∗^*P* < 0.05 was considered to be statistically significant. NS indicated no significance.

## Results

### *Porphyromonas gingivalis* Promotes the Migration and Adhesion of THP-1 Cells to HUVECs

**FIGURE 1:**
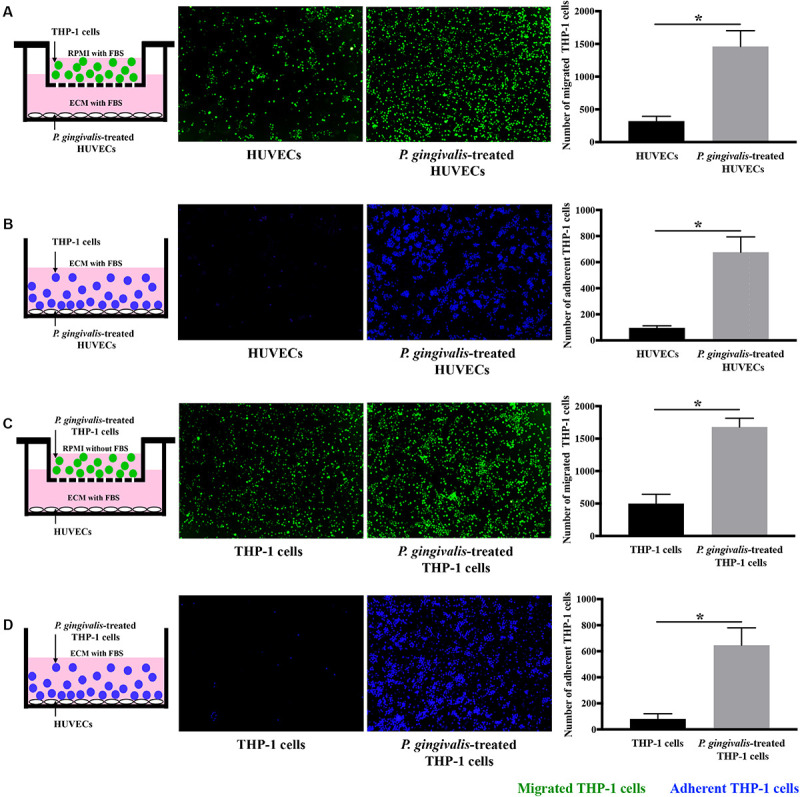
*Porphyromonas gingivalis* promotes THP-1 cell migration and adhesion to HUVECs. **(A)** HUVECs in the lower chambers of transwell systems remained unchallenged or were challenged with *P. gingivalis* for 24 h. Calcein AM-labeled THP-1 cells were loaded into the upper inserts. After incubation for 6 h, the migrated THP-1 cells with green fluorescence were visualized and counted. **(B)** HUVECs in 24-well plates were exposed to *P. gingivalis* for 24 h or untreated. Hoechst 33342-labeled THP-1 cells were added to the HUVECs and incubated for 2 h. After removing the non-adherent THP-1 cells, the adherent THP-1 cells with blue fluorescence were measured. **(C)** THP-1 cells were not treated or treated with *P. gingivalis* for 24 h, labeled with calcein AM, and then loaded into the upper chambers of transwell systems. HUVECs cultured in the lower compartments were cocultured with the THP-1 cells for 6 h. The migrated THP-1 cells were visualized and counted. **(D)** THP-1 cells were challenged with *P. gingivalis* for 24 h or untreated, labeled with Hoechst 33342, and then were added to HUVECs in 24-well plates and incubated for 2 h. The adherent THP-1 cells were imaged and quantified. The quantifications of migrated and adherent THP-1 cells are presented as the mean ± standard deviation (SD) of three independent experiments. All data were analyzed using unpaired two-tailed Student’s *t*-test. *P < 0.05 vs untreated control group.

Cell migration and adhesion assays were performed to detect the effects of *P. gingivalis* on monocyte migration and adhesion to endothelial cells. When HUVECs were challenged with *P. gingivalis* for 24 h, the number of THP-1 cells migrated to the HUVECs markedly increased approximately 4.8-fold (Figure 1A), and the number of THP-1 cells adhered to the HUVECs increased approximately 7-fold (Figure 1B). Similarly, treatment of THP-1 cells with *P. gingivalis* for 24 h also enhanced the migration and adhesion of the THP-1 cells to HUVECs (Figures 1C,D). The number of THP-1 cells that migrated and adhered to HUVECs increased approximately 3.4-fold (Figure 1C) and 8-fold (Figure 1D), respectively. These data demonstrate that the migration and adhesion of THP-1 cells to HUVECs are promoted by *P. gingivalis*.

### *Porphyromonas gingivalis* Promotes the Expression of LOX-1 in Both HUVECs and THP-1 Cells

The exact mechanisms underlying the migration and adhesion of THP-1 cells to HUVECs enhanced by *P. gingivalis* are less well understood. The activation of LOX-1 in cardiovascular cell lineages is highly related with the progress of atherosclerosis including monocyte migration and adhesion (Yoshimoto et al., 2011). Whether LOX-1 was a key mediator regulating *P. gingivalis*-triggered migration and adhesion of THP-1 cells to HUVECs was questioned in this study. Therefore, the effect of *P. gingivalis* on the activation of LOX-1 in HUVECs and THP-1 cells was investigated firstly.

**FIGURE 2:**
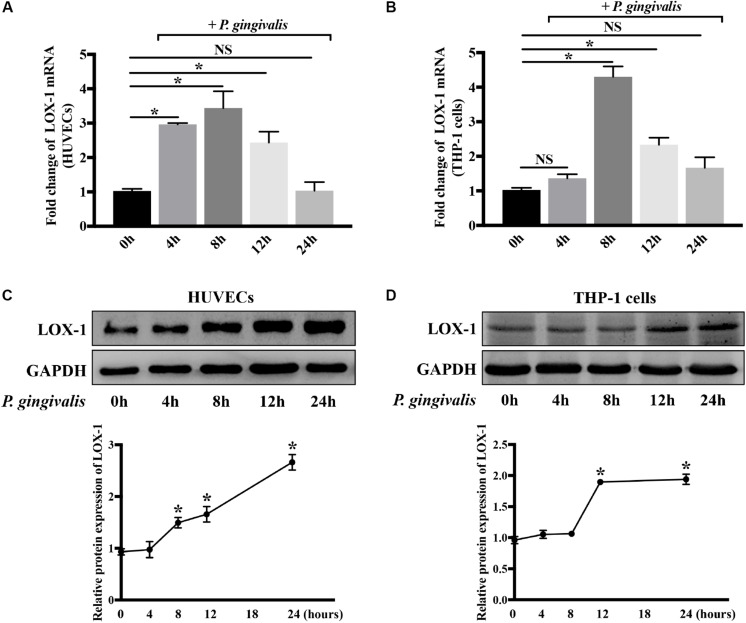
*Porphyromonas gingivalis* increases LOX-1 expression in both HUVECs and THP-1 cells. The mRNA and protein expression levels of LOX-1 in HUVECs **(A,C)** and THP-1 cells **(B,D)** were analyzed after a challenge with *P. gingivalis* at a MOI of 1:100 for 0–24 h. The results are presented as the mean ± SD of four independent experiments. Data were analyzed by one-way analysis of variance (one-way ANOVA) with Dunnett’s multiple comparison. *P < 0.05 vs 0 h.

Both HUVECs and THP-1 cells were treated with *P. gingivalis* (MOI = 100) for 2 h, respectively. Then the medium was replaced with fresh medium and the HUVECs and THP-1 cells continued to be cultured for 0–22 h. Afterward, the expression of LOX-1 in HUVECs and THP-1 cells was analyzed at the mRNA and protein levels. The results showed that LOX-1 expression in both HUVECs and THP-1 cells was low at baseline and increased after the infection of *P. gingivalis* (Figures 2A–D). The mRNA level of LOX-1 in both HUVECs (Figure 2A) and THP-1 cells (Figure 2B) rose to a peak at 8 h. Besides, the protein level of LOX-1 expressed in the HUVECs and THP-1 cells rose to a peak at 24 h (Figures 2C,D, respectively).

### LOX-1 Is Crucially Involved in *P. gingivalis*-Induced THP-1 Cell Migration and Adhesion to HUVECs

**FIGURE 3:**
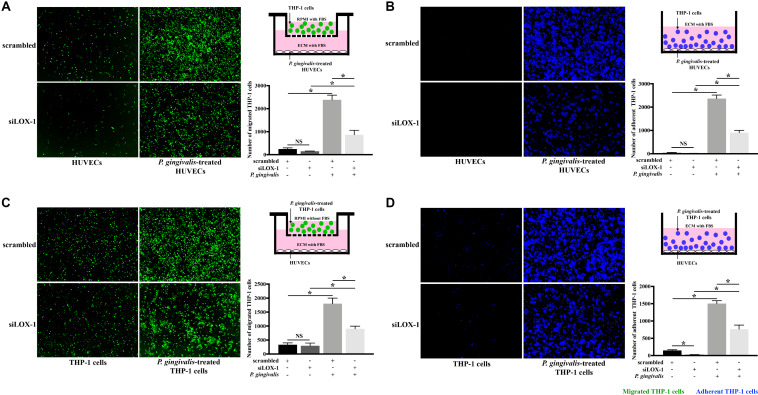
Knockdown of LOX-1 attenuates *P. gingivalis*-induced THP-1 cell migration and adhesion to HUVECs. LOX-1 downregulation by siRNA suppressed LOX-1 expression by about 76–81% in HUVECs and THP-1 cells. **(A)** HUVECs with or without LOX-1 knockdown in the lower compartments of transwell culture dishes remained untreated or were challenged with *P. gingivalis* for 24 h. Calcein AM-labeled THP-1 cells were added into the upper chambers. After 6 h incubation with the HUVECs, THP-1 cells migrating into the lower chambers were imaged and counted. **(B)** HUVECs with or without LOX-1 knockdown in 24-well plates were untreated or treated with *P. gingivalis* for 24 h. They were then cocultured with THP-1 cells (labeled with Hoechst 33342) for 2 h, respectively. The adherent THP-1 cells were measured. **(C)** THP-1 cells with or without LOX-1 knockdown were untreated or challenged with *P. gingivalis* for 24 h before being stained with calcein AM. They were then loaded into the upper chambers of transwell culture dishes separately and incubated with HUVECs cultured in the lower chambers for 6 h. The number of migrated THP-1 cells were counted. **(D)** THP-1 cells with or without LOX-1 knockdown were untreated or treated with *P. gingivalis* for 24 h, and labeled with Hoechst 33342. They were then added, respectively, to HUVECs cultured in 24-well plates and incubated for 2 h. The number of THP-1 cells adhered to the HUVECs was measured under fluorescence microscopy picture. Images are representative of three independent experiments, and the number of migrated or adherent THP-1 cells is presented as the mean ± SD. Data were analyzed by one-way ANOVA with Turkey’s multiple comparison test. *P < 0.05. vs scrambled, siLOX-1, or scrambled + *P. gingivalis* group.

Subsequently, in order to make clear the role of LOX-1 in the *P. gingivalis*-induced THP-1 cell migration and adhesion to HUVECs, LOX-1 expression in HUVECs and THP-1 cells was knocked down by siRNA-mediated gene silencing. The siLOX-1-3 was ensured to be the most effective siRNA for causing a significant (≈76–81%) decrease in LOX-1 expression (Supplementary Figure S1) and was used in this study. Silencing of LOX-1 in HUVECs stimulated by *P. gingivalis* significantly reduced THP-1 cell migration and adhesion to the HUVECs (Figures 3A,B, respectively). Similarly, silencing of LOX-1 in THP-1 cells which were challenged with *P. gingivalis* also reduced migration (Figure 3C) and adhesion (Figure 3D) of the THP-1 cells to HUVECs.

**FIGURE 4:**
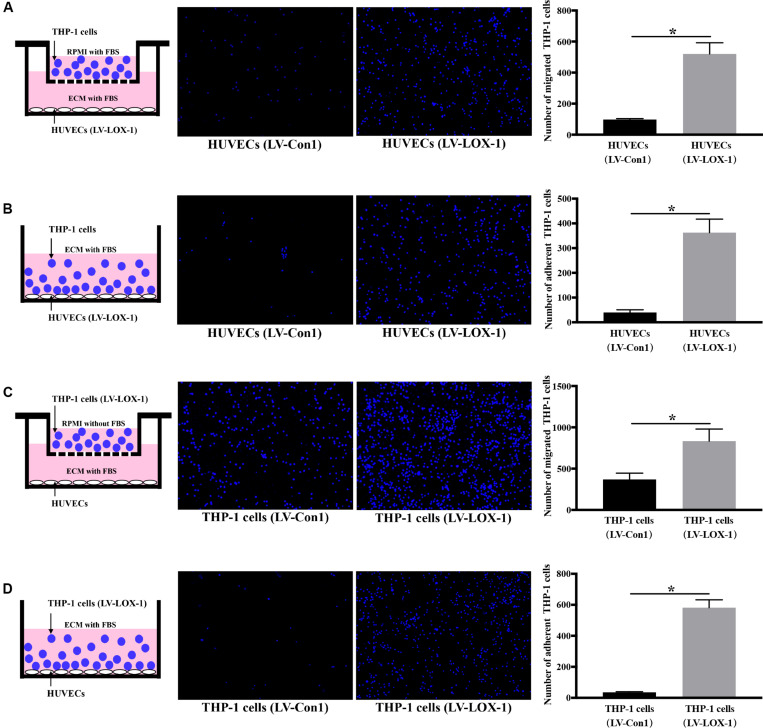
LOX-1 overexpression promotes migration and adhesion of THP-1 cells to HUVECs. LOX-1 in HUVECs and THP-1 cells was overexpressed by about 1.7-fold. **(A)** LOX-1-overexpressing and control HUVECs were cultured in the lower chambers of transwell systems, respectively. THP-1 cells stained with Hoechst 33342 were loaded into the upper chambers and incubated with the HUVECs for 6 h. Migrated THP-1 cells were visualized and counted. **(B)** LOX-1-overexpressing and control HUVECs grown in 24-well plates were cocultured with the Hoechst 33342-labeled THP-1 cells for 2 h, respectively. The non-adherent THP-1 cells were washed, while the adherent THP-1 cells were imaged and quantified. **(C)** LOX-1-overexpressing and control THP-1 cells were labeled with Hoechst 33342 and loaded into the upper inserts of transwell culture dishes separately. HUVECs cultured in the lower chambers were incubated with the THP-1 cells for 6 h. The migrated THP-1 cells were imaged and counted. **(D)** LOX-1-overexpressing THP-1 cells and the control THP-1 cells were labeled with Hoechst 33342, and then added separately to HUVECs cultured in 24-well plates and incubated for 2 h. Adherent THP-1 cells were counted. The number of migrated or adherent THP-1 cells is shown as the mean ± SD (*n* = 4). Data were obtained by unpaired two-tailed Student’s *t*-test. *P < 0.05. vs LV-Con1 group.

To further verify LOX-1 was able to regulate the *P. gingivalis*-induced THP-1 cells migration and adhesion to HUVECs, LOX-1 was overexpressed in HUVECs and THP-1 cells by adenovirus-mediated gene transduction. The effective overexpression of LOX-1 level (increased about 1.7 times) was illustrated as shown in Supplementary Figure S2. The migration (Figures 4A,C) and adhesion (Figures 4B,D) of THP-1 cells to HUVECs were significantly increased by the overexpression of LOX-1 in either HUVECs or THP-1 cells.

These results above show that LOX-1 participates in *P. gingivalis*-mediated THP-1 cell migration and adhesion to HUVECs.

### LOX-1 Regulates *P. gingivalis*-Induced Expression of MCP-1, ICAM-1, and E-Selectin in HUVECs and That of CCR2 and Integrin αMβ2 in THP-1 Cells

The increased expression of ligand/receptor pairs MCP-1/CCR2, ICAM-1/Integrin αMβ2, and E-selectin/Integrin αMβ2 have been known to mediate monocyte migration and adhesion to endothelial cells (Jaipersad et al., 2014; França et al., 2017). Therefore, we detected the influence of LOX-1 on *P. gingivalis*-induced expression of MCP-1, ICAM-1, and E-selectin in HUVECs and that of CCR2 and Integrin αMβ2 in THP-1 cells.

**FIGURE 5:**
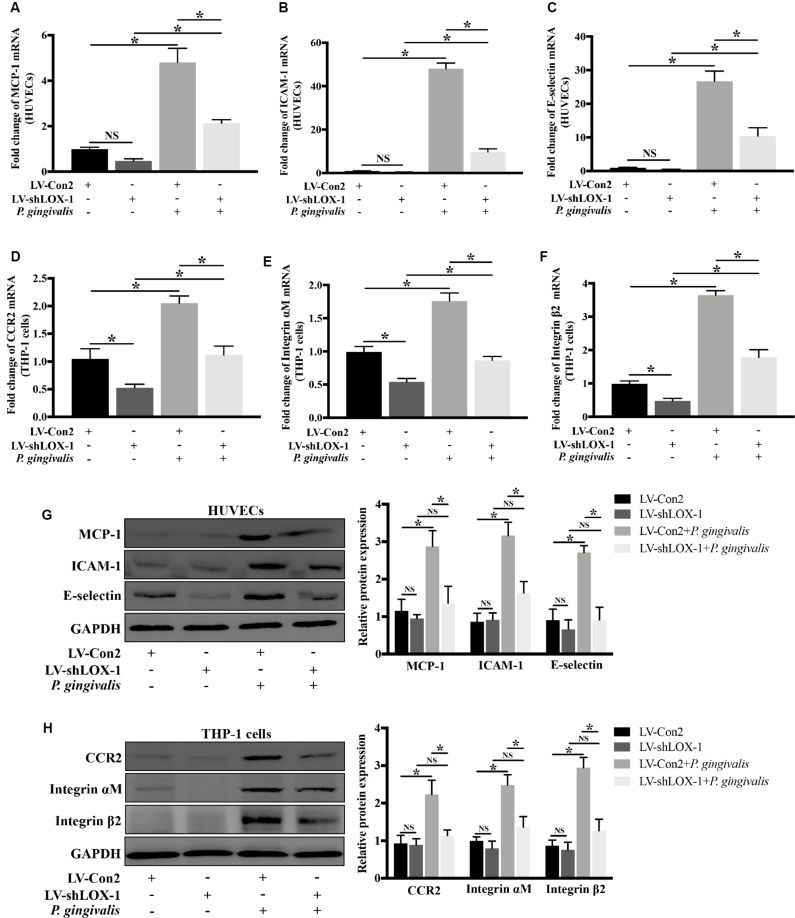
Knockdown of LOX-1 reduces the *P. gingivalis*-induced upregulation of ligands MCP-1, ICAM-1, and E-selectin in HUVECs and that of receptors CCR2 and Integrin αMβ2 in THP-1 cells. Adenovirus-mediated LOX-1 knockdown was achieved in HUVECs and THP-1 cells (knockdown efficiency was about 76%). HUVECs with or without LOX-1 knockdown (LV-shLOX-1 and LV-Con2, respectively) were challenged with *P. gingivalis* for 24 h or untreated. The expression of MCP-1, ICAM-1, and E-selectin was examined by Real-time PCR **(A–C)** and Western blotting **(G)**. THP-1 cells with or without LOX-1 knockdown (LV-shLOX-1 and LV-Con2, respectively) were not treated or treated with *P. gingivalis* for 24 h. The mRNA **(D–F)** and protein **(H)** expression levels of CCR2 and Integrin αMβ2 were also analyzed by Real-time PCR and Western blotting. Results are representative of three independent experiments with similar results ± SD. Data were analyzed using one-way ANOVA with Turkey’s multiple comparison test. *P < 0.05. vs LV-Con2, LV-shLOX-1, or LV-Con2 + *P. gingivalis* group.

Silencing of LOX-1 in HUVECs and THP-1 cells was achieved by lentiviral shRNA transduction. A transduction efficiency of 70–80% was obtained (Supplementary Figures S2A,B). HUVECs and THP-1 cells with or without LOX-1 knockdown (LV-shLOX-1 and LV-Con2, respectively) were challenged with *P. gingivalis* for 24 h or untreated before cells were harvested. The results showed that in HUVECs, the mRNA (Figures 5A–C) and protein (Figure 5G) expression levels of ligands MCP-1, ICAM-1, and E-selectin, especially ICAM-1 and E-selectin were upregulated strongly by *P. gingivalis*. However, LOX-1 silencing dramatically repressed the upregulation (Figures 5A–C,G). Likewise, in THP-1 cells, stimulation with *P. gingivalis* also resulted in an increase in the expression of receptors CCR2, Integrin αM and Integrin β2, and the increase was substantially reduced by knockdown of LOX-1 (Figures 5D–F,H).

**FIGURE 6:**
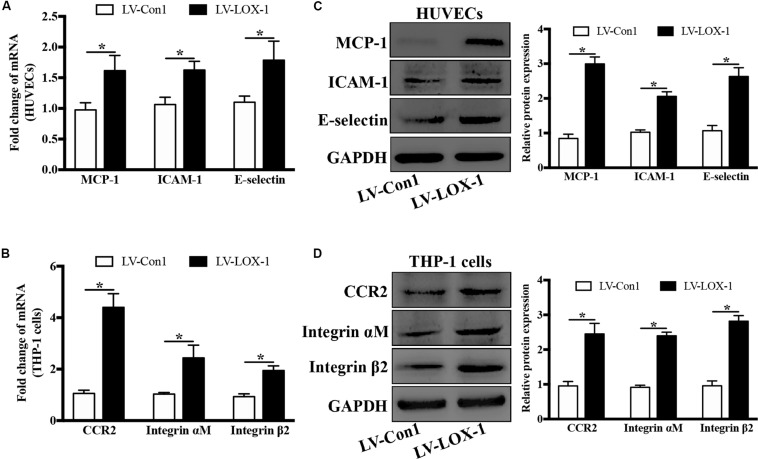
LOX-1 overexpression increases expression of ligands MCP-1, ICAM-1, and E-selectin in HUVECs and that of receptors CCR2 and Integrin αMβ2 in THP-1 cells. LOX-1 expression in HUVECs and THP-1 cells was enhanced by about 1.7-fold by lentiviral transduction. The mRNA **(A)** and protein **(C)** expression levels of MCP-1, ICAM-1, and E-selectin in LOX-1-overexpressing and control (LV-LOX-1 and LV-Con1, respectively) HUVECs were detected by Real-time PCR and Western blotting, respectively. The same methods were also adopted to examine the mRNA **(B)** and protein **(D)** expression levels of CCR2 and Integrin αMβ2 in LOX-1-overexpressing (LV-LOX-1) and control (LV-Con1) THP-1 cells. The results are representative of three independent experiments and are presented as the mean ± SD. Data were analyzed using unpaired two-tailed Student’s *t*-test. *P < 0.05. vs LV-Con1 group.

To verify the effect of LOX-1 on the expression of ligands MCP-1, ICAM-1, and E-selectin in HUVECs and that of receptors CCR2 and Integrin αMβ2 in THP-1 cells, LOX-1 was overexpressed in HUVECs and THP-1 cells (increased by about 1.7-fold). It was found that the mRNA (Figure 6A) and the protein (Figure 6C) expression levels of MCP-1, ICAM-1 and E-selectin in HUVECs were elevated by LOX-1 overexpression. Meanwhile, LOX-1 overexpression also caused a increase in the mRNA (Figure 6B) and protein (Figure 6D) presentation of CCR2 and Integrin αMβ2 in THP-1 cells.

These results demonstrate that LOX-1 plays a positive role in *P. gingivalis*-induced expression of MCP-1, ICAM-1 and E-selectin in HUVECs and that of CCR2 and Integrin αMβ2 in THP-1 cells.

### Induction of LOX-1 by *P. gingivalis* Is Dependent on NF-κB Activation

**FIGURE 7:**
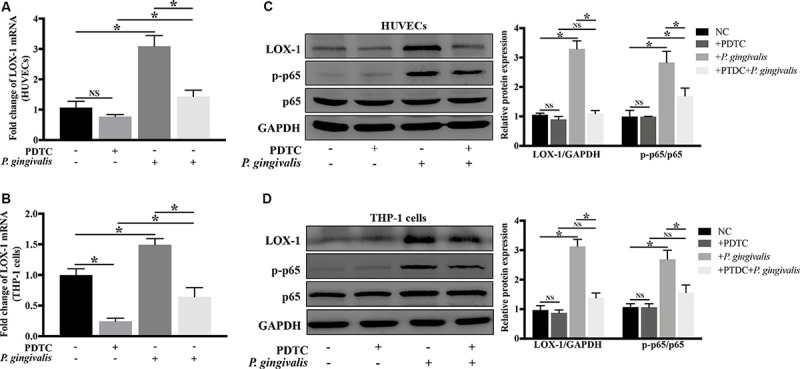
Blockage of NF-κB signaling abrogates *P. gingivalis*-induced LOX-1 production in HUVECs and THP-1 cells. HUVECs and THP-1 cells were pretreated with PDTC (100 μM, an inhibitor of NF-κB activation) or not treated before they were challenged with *P. gingivalis* or left unchallenged. The inhibitory effect of PDTC on NF-κB activation in HUVECs **(C)** and THP-1 cells **(D)** was evaluated by Western blotting. Meanwhile, the effect of PDTC on *P. gingivalis*-upregulated LOX-1 expression in HUVECs **(A,C)** and THP-1 cells **(B,D)** was examined. Data represents average ± SD (*n* = 4). Statistical differences were determined using one-way ANOVA followed by Turkey’s multiple comparison test. *P < 0.05. vs normal control cells (NC), + PDTC, or + *P. gingivalis* group.

NF-κB as a crucial transcription factor, is involved in response to various stimuli such as bacterial infections and inflammatory factors. In order to evaluate whether NF-κB signaling pathway mediates the activation of LOX-1 in HUVECs and THP-1 cells induced by *P. gingivalis*, HUVECs and THP-1 cells were preincubated with PDTC (100 μM) for 1 h to inhibit the activation of NF-κB, then followed by *P. gingivalis* stimulation for another 24 h before cells were harvested. The results of Real-time PCR showed that the mRNA level of LOX-1 in both HUVECs and THP-1 cells increased significantly after *P. gingivalis* stimulation, but the increase was inhibited by PDTC pretreatment (Figures 7A,B). Western blotting revealed that *P. gingivalis* stimulation led to an increased expression of p–p65, which was markedly suppressed by PDTC preincubation (Figures 7C,D). Moreover, the increased protein expression of LOX-1 in both HUVECs and THP-1 cells by *P. gingivalis* stimulation was also inhibited substantially by PDTC (Figures 7C,D). These results demonstrate that the NF-κB signal pathway mediates the LOX-1 expression in HUVECs and THP-1 cells induced by *P. gingivalis*.

### LOX-1 Modulates the Production of MCP-1, ICAM-1, and E-Selectin in HUVECs and That of CCR2 and Integrin αMβ2 in THP-1 Cells via NF-κB Activation

**FIGURE 8:**
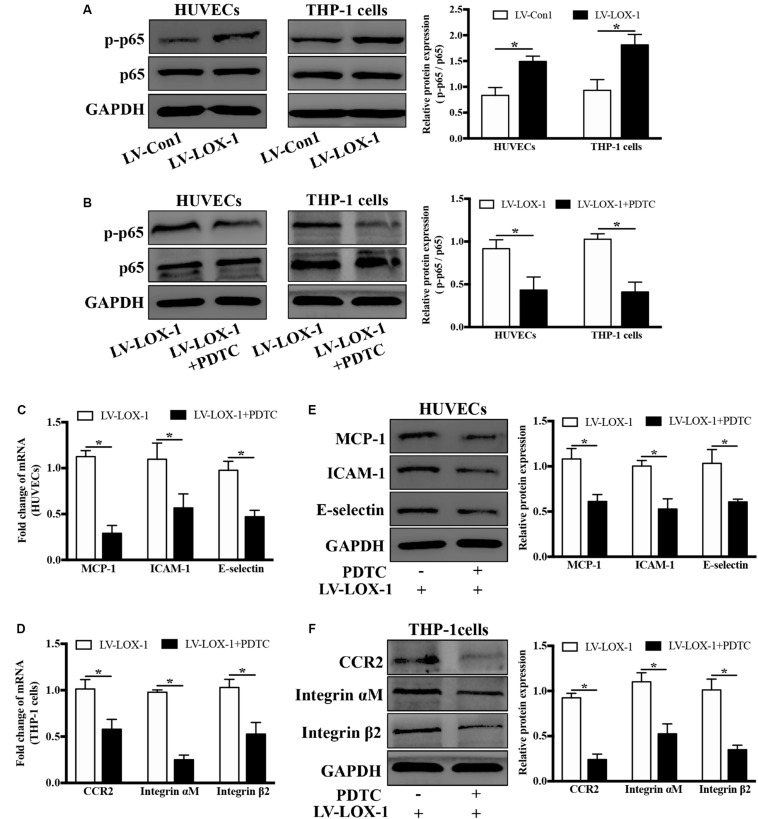
Blockage of NF-κB signaling attenuates LOX-1-upregulated expression of MCP-1, ICAM-1, and E-selectin in HUVECs and that of CCR2 and Integrin αMβ2 in THP-1 cells. **(A)** The expression levels of p–p65 and p65 in LOX-1-overexpressing HUVECs, LOX-1-overexpressing THP-1 cells, and their corresponding control groups were detected by Western blotting. **(B)** PDTC (100 μM) was applied to block the activation of NF-κB in LOX-1-overexpressing HUVECs and LOX-1-overexpressing THP-1 cells. After inhibition of NF-κB signaling, the expression change of MCP-1, ICAM-1, and E-selectin in LOX-1-overexpressing HUVECs **(C,E)** and that of CCR2 and Integrin αMβ2 in LOX-1-overexpressing THP-1 cells **(D,F)** were evaluated. Data are mean ± SD (*n* = 4), which were analyzed by unpaired two-tailed Student’s *t*-test. *P < 0.05. vs LV-Con1 or LV-LOX-1 group.

Next, the regulatory role of NF-κB signaling pathway in LOX-1-dependent induction of MCP-1, ICAM-1 and E-selectin in HUVECs and that of CCR2 and Integrin αMβ2 in THP-1 cells was investigated. As shown in Figure 8A, there was a higher level of p–p65 expression in LOX-1-overexpressing HUVECs and LOX-1-overexpressing THP-1 cells than that in their control groups, which indicated the activation of NF-κB signaling pathway by LOX-1. Therefore, PDTC (100 μM, 24 h) was used to inhibit the NF-κB activation, and the p–p65 expression in both LOX-1-overexpressing HUVECs and LOX-1-overexpressing THP-1 cells was considerably decreased by PDTC treatment (Figure 8B). In addition, the mRNA expression of MCP-1, ICAM-1 and E-selectin in LOX-1-overexpressing HUVECs was significantly attenuated by PDTC (Figure 8C). This attenuation was verified at the protein level (Figure 8E). The mRNA (Figure 8D) and protein (Figure 8F) expression of CCR2 and Integrin αMβ2 in LOX-1-overexpressing THP-1 cells was decreased as well by treatment with PDTC. These results reveal that NF-κB signaling is involved in LOX-1-induced expression of MCP-1, ICAM-1 and E-selectin in HUVECs and that of CCR2 and Integrin αMβ2 in THP-1 cells.

## Discussion

Monocyte migration and adhesion to endothelial cells are crucial steps in the early stages of atherosclerosis development, in which ligand-receptor pairs such as MCP-1/CCR2, ICAM-1/Integrin αMβ2, and E-selectin/Integrin αMβ2 are crucially involved. The significant finding of this study is that LOX-1 is crucially involved in *P. gingivalis*-induced monocyte migration and adhesion to endothelial cells, by regulating the expression of not only the chemokine MCP-1, and adhesion molecules ICAM-1, and E-selectin in HUVECs but also their corresponding receptors CCR2 and Integrin αMβ2 in THP-1 cells.

It was demonstrated for the first time that LOX-1 in both HUVECs (Figures 2A,C) and THP-1 cells (Figures 2B,D) could be activated by the periodontal pathogen *P. gingivalis*. Previous studies have reported that LOX-1 recognized some pathogens such as *S. aureus*, *E. coli* (Shimaoka et al., 2001), *C. pneumoniae* (Campbell et al., 2013) and *Aspergillus* (He et al., 2016). However, no studies have focused on the response of LOX-1 to *P. gingivalis*. *P. gingivalis* is a keystone periodontal pathogen. A close relationship between *P. gingivalis* and cardiovascular disease has been suggested. This pathogen could translocate from periodontal pockets to the cardiovascular system (Spahr et al., 2006). *P. gingivalis* was even found in human atheroma (Fiehn et al., 2005; Mahendra et al., 2015) and cells from atherosclerotic lesions (Njoroge et al., 1997; Olsen and Progulske-Fox, 2015). LOX-1 is widely known to play a vital role in atherosclerosis (Singh and Gautam, 2019). This receptor has been proved to contribute to the formation of atherosclerotic lesions in the initiation of atherosclerosis. Furthermore, the persistent accumulation of the LOX-1 is also involved in atherosclerotic plaque stability and myocardial infarction, which implies the long-term effects of LOX-1 in atherosclerosis (Chen et al., 2002). In the present study, periodontal pathogen *P. gingivalis* was found to increase the expression level of LOX-1 in both HUVECs (Figures 2A,C) and THP-1 cells (Figures 2B,D). The upregulation of LOX-1 expression by *P. gingivalis* supports the important role of LOX-1 in *P. gingivalis*-mediated pathological processes in atherosclerosis and reinforces the speculation that LOX-1 is a potential molecule linking periodontitis with atherosclerosis.

Previous studies have suggested that LOX-1 was absent in monocytes but induced in differentiated macrophages (Yoshida et al., 1998; Noriaki et al., 2006). However, our results were not in line with those results. In our study, the expression of LOX-1 in monocytic THP-1 cells was low, but it was increased significantly by *P. gingivalis* stimulation (Figures 2B,D). More recently, Tanimoto (Tanimoto et al., 2001) and Yamagata (Yamagata et al., 2014) revealed that LOX-1 expression in unstimulated monocytes was low, which was consistent with our results. These two research teams also detected upregulated LOX-1 expression in monocytes which were stimulated with histamine and TNF-α, respectively (Tanimoto et al., 2001; Yamagata et al., 2014). Their results were similar to our results that LOX-1 in THP-1 cells was inducible by some external stimuli. In addition, the production of LOX-1 has been demonstrated to be remarkably high in monocytes from patients with acute coronary syndrome, compared with those from healthy controls (Wang et al., 2008). Hence, it is concluded that LOX-1 is actually expressed at a low level in normal monocytes but it is highly expressed in activated monocytes.

Surprisingly, *P. gingivalis* triggered a similar kinetics of LOX-1 expression in HUVECs and THP-1 cells, in our study. Although LOX-1 in THP-1 cells seemed to exhibit a more limited response to *P. gingivali*s than that in HUVECs, the mRNA and protein levels of LOX-1 expression in both cell lines peaked at 8 h and 24 h, respectively, under the same stimulation of *P. gingivalis* (Figures 2A–D). To our knowledge, for the first time the kinetics of LOX-1 expression in HUVECs and THP-1 cells is compared. Endothelial cells and monocytes are thought to coordinately regulate some specific immune processes such as monocyte migration and adhesion (Chanput et al., 2014), in which LOX-1 plays a vital role. The similar kinetics of LOX-1 expression in HUVECs and THP-1 cells is possibly the premise and foundation for synchronous cooperation between these two cell lines, finally guiding THP-1 cell migration and adhesion to HUVECs. Moreover, it was of interest to note that LOX-1 induction by *P. gingivali*s in both HUVECs and THP-1 cells was regulated by NF-κB pathway (Figures 7A–D). NF-κB activation is considered to be a central initiating cellular event in response to a wide range of pathogens (Rahman and McFadden, 2011). As the full spectrum of NF-κB-dependent genes are being gradually systematically explored, the sequential expression pattern of NF-κB-downstream genes is revealed (Tian et al., 2005; O’Dea and Hoffmann, 2010). LOX-1 expression represented in a form of quantitative accumulation and rose to a peak at 8 h or even later after *P. gingivali*s stimulation (Figures 2A–D). Thus LOX-1 can be classed into the late response group of NF-κB-downstream genes (Tian et al., 2005). Given that the kinetics of NF-κB activation is a central regulator of its effect on downstream genes (Sen and Smale, 2009; O’Dea and Hoffmann, 2010), it is suggested that HUVECs and THP-1 cells share a similar activation pattern of NF-κB for the similar kinetics of LOX-1 expression. This possibility requires further research. In conclusion, the similar kinetics of LOX-1 expression via NF-κB activation in HUVECs and THP-1 cells may be the possible foundation for their synchronous cooperation, which determines the migration and adhesion of THP-1 cells to HUVECs. This postulation inspires us to explore the effect of LOX-1 on monocyte-endothelial cell communication in a new dimension.

Moreover, it is worth noting that LOX-1 was involved in the regulation of not only ligands MCP-1, ICAM-1 and E-selectin expression in HUVECs but also corresponding receptors CCR2 and Integrin αMβ2 expression in THP-1 cells (Figures 6A–D). The binding of chemokines and adhesion molecules in HUVECs to their counterreceptors in monocytes are importantly implicated in monocyte migration and adhesion to HUVECs (Miller and Blystone, 2015). Monocyte chemoattractant protein-1 (MCP-1) is one of the most important regulators, whose properties of monocyte activation and recruitment are primarily mediated through the activation of CCR2 (França et al., 2017). Monocyte adhesion to endothelial cells fosters atherosclerotic lesion initiation. In this process, the interaction of E-selectin/Integrin αMβ2 mediates monocyte rolling on endothelial cells and monocyte adhesion to endothelial cells, and the interaction of ICAM-1/Integrin αMβ2 mainly determines the firm adhesion of monocytes (Jaipersad et al., 2014). The activation of various migration-related and adhesion-related molecules in our study emphasizes the importance of LOX-1 in monocyte migration and adhesion to endothelial cells. Besides, the dual regulatory role of LOX-1 in activating ligands in HUVECs and corresponding receptors in THP-1 cells, compared with the activation of ligands or receptors alone, would enhance the affinity between these two cell lines, further facilitating monocyte migration and adhesion to endothelial cells. More interestingly, *P. gingivalis* as an effective activator for LOX-1, was also found to upregulate the expression of the chemokine MCP-1, adhesion molecules ICAM-1 and E-selectin in HUVECs and that of their receptors CCR2 and Integrin αMβ2 in THP-1 cells, an effect was substantially reduced following LOX-1 knockdown (Figures 5A–D). Thus, these evidences prominently emphasize the importance of LOX-1 in *P. gingivalis*-induced monocyte migration and adhesion of monocytes to endothelial cells, by modulating migration-related and adhesion-related molecules.

NF-κB is generally known to be associated with inflammatory diseases, such as periodontitis, atherosclerosis and type 2 diabetes (Baker et al., 2011; Hajishengallis and Sahingur, 2014). More importantly, NF-κB has a central role in inflammatory network involving complicated cross talks and feedbacks (Tieri et al., 2012; Hu et al., 2017). Our results revealed that induction of LOX-1 by *P. gingivalis* was dependent on NF-κB activation (Figures 7A–D). The promoter region of the LOX-1 gene was reported to contain a binding site for NF-κB (Kelly et al., 2008). This important discovery supports our result that NF-κB signaling pathway plays an important role in LOX-1 activation. The ligand-receptor pairs MCP-1/CCR2, ICAM-1/Integrin αMβ2, and E-selectin/Integrin αMβ2 are important molecules for monocyte migration and adhesion to endothelial cells (Jaipersad et al., 2014; França et al., 2017). We found that NF-κB signaling pathway also mediate LOX-1-dependent expression of MCP-1, ICAM-1 and E-selectin in HUVECs, and that of their receptors CCR2 and Integrin αMβ2 in THP-1 cells (Figures 8A–F). This result indicated that NF-κB is an important signaling pathway for LOX-1-downstream genes. Based on these results, it is so interesting to note that NF-κB-dependent LOX-1 regulates downstream molecules through NF-κB activation as well. We suggest that the central role of NF-κB/LOX-1 positive feedback loop is the main reason. Specifically, *P. gingivali*s-activated NF-κB signaling result in the induction of LOX-1, which subsequently induces NF-κB activation, thereby creating a positive feedback loop. The loop can further serve as an “inflammation amplifier”, whose role is to further enhance the presentation of downstream migration-related and adhesion-related molecules. In this interaction mode, NF-κB acts as a key node regulating the expression of LOX-1 and LOX-1-dependent downstream molecules. Similar interaction patterns are found in other researches, in which molecules achieving self-regulation through NF-κB activation can regulate other NF-κB-dependent responses at the same time (Hosokawa et al., 2013; Ogura et al., 2013; Zhu et al., 2019). Collectively, our results highlight the central role of NF-κB/LOX-1 positive feedback loop in the expression of ligands MCP-1, ICAM-1, and E-selectin in HUVECs, and that of receptors CCR2 and Integrin αMβ2 in THP-1 cells.

Altogether, the results of this study demonstrate an essential involvement of LOX-1 in *P. gingivalis-*triggered monocyte migration and adhesion to endothelial cells by regulating the presentation of the chemokine MCP-1, adhesion molecules ICAM-1 and E-selectin in HUVECs and that of their receptors CCR2 and Integrin αMβ2 in THP-1 cells. These evidences *in vitro* deepen our understanding of the molecular mechanism involved in *P. gingivalis*-induced monocyte migration and adhesion to endothelial cells. In addition, a potential role for LOX-1 in the link between periodontitis and atherosclerosis is preliminarily verified.

## Data Availability Statement

All datasets generated for this study are included in the article/Supplementary Material.

## Author Contributions

QL designed and performed the research, collected and analyzed the data, and wrote the manuscript. JL and WL performed the research, collected the data, and edited the manuscript. YC, JZ, YX, and XL performed the research, collected the data, and provided analysis tools. XO designed the research, supported financial support for the research, and edited the manuscript. All authors read and approved the manuscript.

## Conflict of Interest

The authors declare that the research was conducted in the absence of any commercial or financial relationships that could be construed as a potential conflict of interest.

## Supplementary Material

FIGURE S1 Efficiency of LOX-1 knockdown in HUVECs and THP-1 cells. LOX-1 knockdown in HUVECs **(A)** and THP-1 cells **(B)** was achieved with a small interfering RNA (siLOX-1). Their transfection efficiency was determined by Western blotting. GAPDH was used for normalization. The results are presented as the mean ± SD of three independent experiments. The statistical significance was measured by one-way ANOVA and Dunnett’s multiple comparison tests. ^∗^*P* < 0.05. vs scrambled group.

FIGURE S2 Generation of LOX-1-overexpressing and LOX-1-knockdown cell lines. Stable overexpression or knockdown of LOX-1 (LV-LOX-1 and LV-shLOX-1, respectively) in HUVECs **(A)** and THP-1 cells **(B)** was achieved by lentiviral infection. Their efficiencies were determined by Western blotting. Data shown are the mean ± SD (*n* = 3). Statistical differences were determined by unpaired two-tailed Student’s *t*-test. ^∗^*P* < 0.05. vs LV-Con1 or LV-Con2 group.

## Abbreviations

CCR2: C–C chemokine receptor 2
HUVECs: human umbilical vein endothelial cells
ICAM-1: intercellular adhesion molecule-1
LOX-1: lectin-like oxidized low-density lipoprotein receptor-1
LV-Con1: control of LOX-1 overexpression
LV-LOX-1: LOX-1 overexpression
LV-shLOX-1: control of LOX-1 knockdown
LV-shLOX-1: LOX-1 knockdown
MCP-1: monocyte chemoattractant protein-1
MOI: multiplicity of infection
NF- κ B: nuclear factor-kappa B
ox-LDL: oxidized low-density lipoprotein
*P. gingivalis*: *Porphyromonas gingivalis*
PDTC: ammonium pyrrolidinedithiocarbamate
PRR: pattern recognition receptor
PVDF: polyvinylidenedifluoride
siRNA: small interfering RNA
SMC: smooth muscle cells
TGF- β: transfer growth factor β
TNF- α: Tumor necrosis factor α.
