# Supplementary figures and images for: LOX-1 Regulates P. gingivalis-Induced Monocyte Migration and Adhesion to Human Umbilical Vein Endothelial Cells
Source: Front Cell Dev Biol. 2020 Jul 14;8:596. doi: 10.3389/fcell.2020.00596 (PMC7394702; doi:10.3389/fcell.2020.00596)

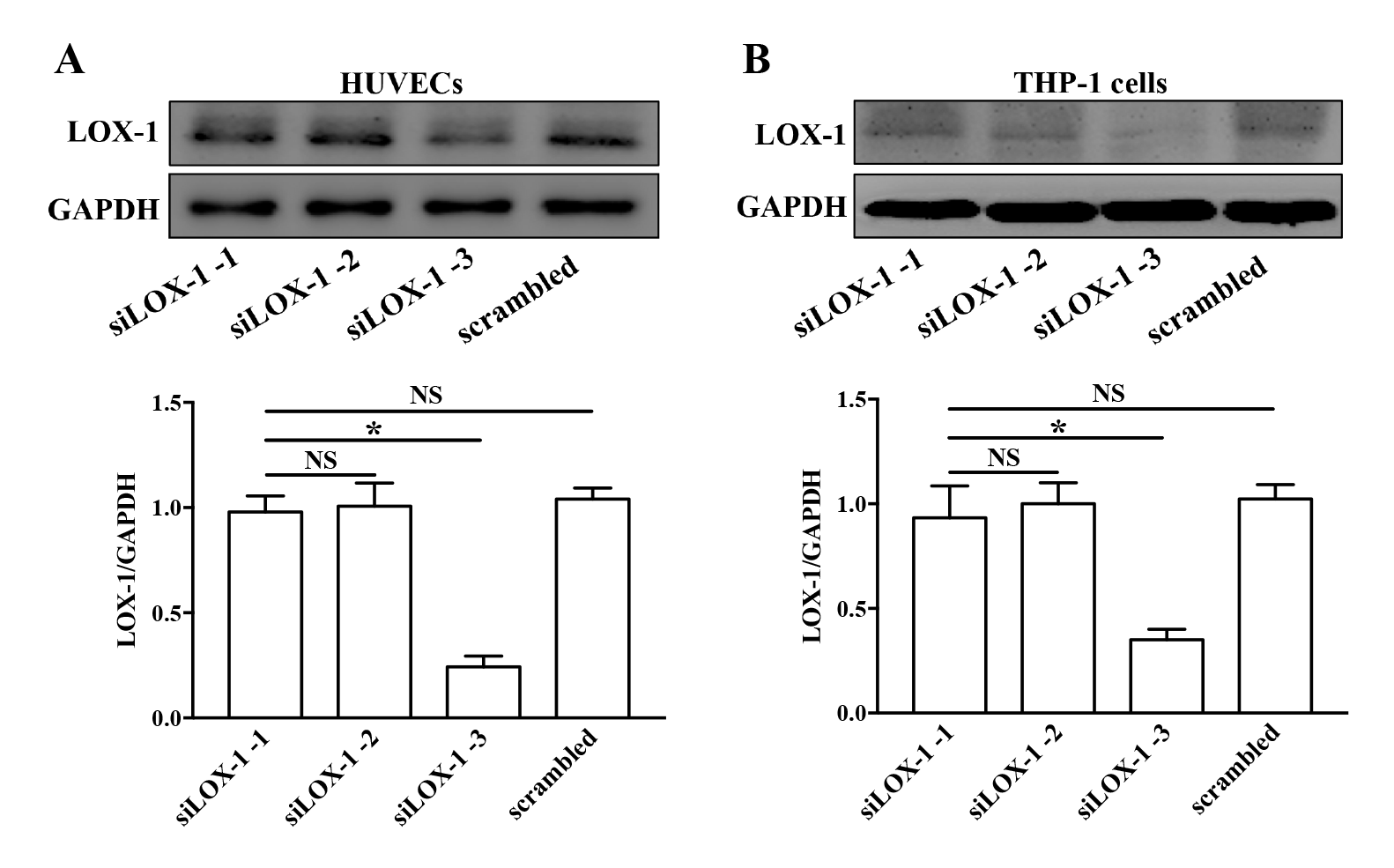

Supplement: FIGURE S1 — Efficiency of LOX-1 knockdown in HUVECs and THP-1 cells. LOX-1 knockdown in HUVECs (A) and THP-1 cells (B) was achieved with a small interfering RNA (siLOX-1). Their transfection efficiency was determined by Western blotting. GAPDH was used for normalization. The results are presented as the mean ± SD of three independent experiments. The statistical significance was measured by one-way ANOVA and Dunnett’s multiple comparison tests. ∗P < 0.05. vs scrambled group. [file Image_1.TIF]

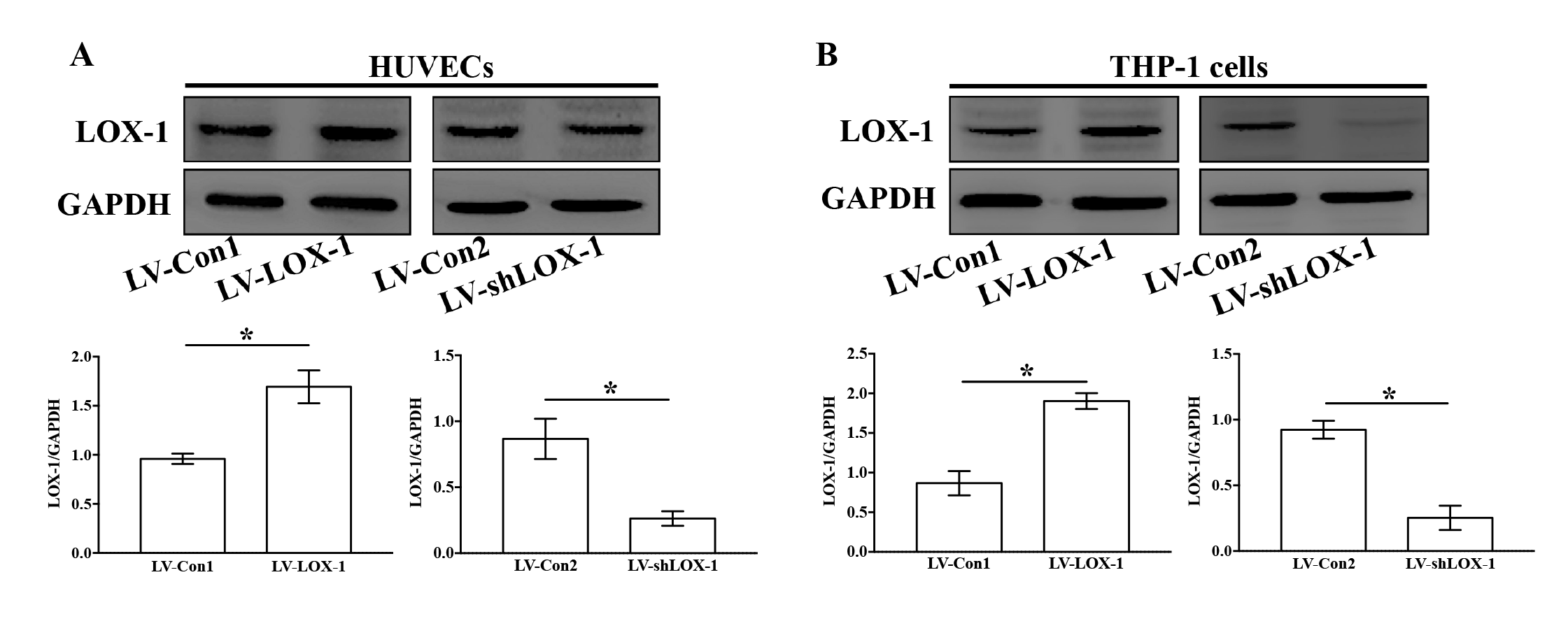

Supplement: FIGURE S2 — Generation of LOX-1-overexpressing and LOX-1-knockdown cell lines. Stable overexpression or knockdown of LOX-1 (LV-LOX-1 and LV-shLOX-1, respectively) in HUVECs (A) and THP-1 cells (B) was achieved by lentiviral infection. Their efficiencies were determined by Western blotting. Data shown are the mean ± SD (n = 3). Statistical differences were determined by unpaired two-tailed Student’s t-test. ∗P < 0.05. vs LV-Con1 or LV-Con2 group. [file Image_2.TIF]
